# Personnel and Participant Experiences of a Residential Weight-Loss Program. A Qualitative Study

**DOI:** 10.1371/journal.pone.0100226

**Published:** 2014-06-17

**Authors:** Unni Dahl, Marit By Rise, Bård Kulseng, Aslak Steinsbekk

**Affiliations:** 1 Central Norway Health Authority, Stjørdal, Norway; 2 Department of Public Health and General Practice, Norwegian University of Science and Technology, Trondheim, Norway; 3 Regional Centre for Obesity Treatment, St. Olav’s University Hospital, Trondheim, Norway; The University of New South Wales, Australia

## Abstract

**Background:**

Residential weight-loss programs aim to help persons with obesity lose weight and maintain a long-term healthy lifestyle. Knowledge is needed on the different actors’ perceptions and experiences from such programs. The aim of this study was to describe how personnel argued for and perceived a residential weight-loss program, to investigate how the participants experienced the program, and to contrast these perspectives.

**Methods:**

This qualitative study took place in an 18-week residential weight-loss program. Exercise, diet, and personal development were the main components in the program. Data was collected through participant observation and individual and focus group interviews with participants and personnel.

**Results:**

Program personnel characterized persons with obesity in specific terms, and these formed the basis of the educational aims, teaching principles, and content of the program. According to personnel, persons with obesity typically had problems acknowledging their own resources, lived unstructured lives, had a distorted relationship to food, experienced a range of social problems and featured a lack of personal insight. Program participants reported enthusiasm about their experiences of exercise and appreciated measures of success with the exercise program. They had, however, very different experiences regarding the usefulness and appropriateness of the parts of the program focused on social and personal development. Some felt that weight loss required an engagement with personal development while others viewed it as unnecessary and inappropriate.

**Conclusion:**

The reliance in personnel accounts on particular characteristics of persons with obesity as a rationale for the program might lead to stigmatizing and stereotyping. Program activities focused on social and personal development need to be better understood by participants if they are to be viewed as helpful. To achieve this personnel must carefully consider how these parts of the program are communicated and conducted.

## Introduction

Obesity is a growing problem in the Western world [1], and many diseases and illnesses accompany it [2], [3]. Recently surgery has become more common to achieve weight-loss. A new systematic review and meta-analysis on the outcomes and risks of bariatric surgery showed that surgery was more effective than non-surgical interventions regarding weight-loss [4]. Although risks of complications exist, death rates were lower than in previous meta-analyses. Meta-analysis of observational studies also indicates that bariatric surgery might lead to a reduced risk of cardiovascular disease and mortality for persons with obesity [5], but this has yet to be investigated in clinical trials. Martins and colleagues compared bariatric surgery to three conservative treatments - including the weight-loss camp that served as the setting for this qualitative study [6]. They found that although all treatments led to significant weight-loss, patients who had undergone bariatric surgery had lost significantly more weight one year after the interventions.

Although weight-loss surgery is increasingly used, the combination of exercise, diet, and behaviour therapy in comprehensive weight-loss programs seems to be the most commonly available intervention [7],[8]. Although many clinical studies of weight-loss programs show that the participants lose weight during and after the intervention [9]–[13], some authors have stated that non-surgical treatments of obesity are generally ineffective in long-term weight-control [4]. One study investigating the long-term maintenance of weight-loss after an extensive lifestyle intervention - at the weight-loss camp investigated in the qualitative study presented here - showed for example that a 15% weight reduction after the intervention was reduced to 5.3% reduction of the initial body weight 2–4 years afterwards [12]. Thus, one of the main challenges in weight-loss programs is to achieve long-term sustainable changes.

Soderlund and colleagues [8] conducted a systematic review of randomized controlled trials investigating long-term effects of physical exercise with or without behavior-change therapy and/or dieting. The authors conclude that the programs with the best results involve different professional groups, and include a combination of diet, behavioral-modification therapy, and exercise. Other studies have also shown that adding exercise or behavior therapy to diet improved weight-loss and reduced the health risk factors connected to obesity [9], [11], [14].

Weight-loss programs thus aim to help the participants modify their health behaviors. The main argument for residential programs is to fortify a sustainable long-term lifestyle change. Residential weight-loss programs constitute a rather new approach [7], and research on the effects of such programs on adults is scarce [15]. Weight-loss programs include behaviour therapies that aims to help the participants achieve the necessary skills to reach a more healthy weight [16]. Lifestyle interventions usually include many behavioural techniques such as self-monitoring, modelling, environmental restructuring, and support, both in groups and individually [13]. Other central terms are self-regulation, goal setting, and problem solving [17], [18]. This makes it difficult to recognize the particular elements which might be supporting learning and behavioural changes. In addition, most behaviour therapy interventions are poorly defined and described in the literature [8]. Many authors have emphasized that interventions in general often are implemented without a deep understanding of how different initiatives are supposed to lead to change [19]. More detailed knowledge about the content of weight-loss programs is therefore required, as well as a deeper understanding of what really happens in lifestyle interventions for persons with obesity.

A residential program differs from out-patient intervention by length and intensity, and in that the participants are removed from their everyday lives. A residential program also offers more time for communication between participants and personnel [20]. Therefore, residential weight-loss programs are arenas for close interaction between participants and personnel, and with a broad range of opportunities provided for personnel to teach and support participants in their endeavour to change behaviour. Although lifestyle-modification programs often combine exercise, diet and behaviour change activities, a good relationship that allows for open communication is generally recommended [21]. Several have also emphasized the importance of motivation to make lifestyle changes [8], [17], [18]. Since motivation to alter lifestyle is crucial to achieve long-term changes, support and help from personnel during such programs would be important [8]. Many have argued that personnel skills and attitudes should be characterized by trust, respect and acceptance when caring for persons with chronic diseases [22], [23]. Respect has been described as unconditionally recognizing the patient as a valuable person, and as an integral part of health professionals’ work [24].

Very little research has explored different actors’ perceptions of and experiences from weight-loss programs. It has been emphasized that to achieve good results different stakeholders in an intervention should share an understanding of how change is supposed to be implemented and sustained [25]. Including both personnel and participants in the same study would contribute to the understanding of the stakeholders’ rationale for an intervention, as well as their views on potential outcome. Therefore, the aim of this study was to describe how personnel argued for and perceived a residential weight-loss program, to investigate how the participants experienced the program, and to contrast these perspectives.

## Methods

### Ethics Statement

The Regional Ethics Committee for Research in Medicine in Central Norway approved of the study. All participants received oral and written information about the study and signed a written consent form. None of the authors have any contact with or interest in the Danish weight-loss camp outside this research project.

### Design

This was a qualitative study consisting of on-site participant observation, in-depth semi-structured individual interviews, and focus group interviews. A combination of methods was chosen to ensure data triangulation. Observation was chosen to provide data on real life interaction between participants and personnel and to gain insight into the participants’ immediate reactions to the program. Focus group interviews were conducted to explore the interaction and discussions between participants regarding their experiences and views of the program. Individual interviews were also employed to explore in more depth the ways in which personnel articulated their arguments for and experiences of providing the program, and to delve deeper into the individual experiences of a selected subset of participants.

The study formed part of a Norwegian clinical investigation on the effectiveness of different interventions (weight-loss program versus surgery) for persons with obesity. The study was registered in Clinicaltrials.gov with registration number NCT00239850. An effect study from this program is published elsewhere [6].

### Setting

The study took place at a Danish residential weight-loss centre (Ebeltoft kurcenter), which offers an 18-week on-site program. A total of 80 participants were at the centre at the time of the data collection. This study focused on 30 participants who were referred to this program due to taking part in a Norwegian study on the effect of different weight-loss approaches [6].

Most of the activities at the centre were organized and conducted in groups. The program consisted of group-based intensive exercise, diet (individual calorie intake was based on energy calculations for a normal weight person with a sedentary activity level), and an educational program. Structured group exercise and educational sessions took place from Monday to Friday every week. Exercise was compulsory three times per day. Typical exercise activities were badminton, ball games, aerobics, and swimming. In addition, participants were encouraged to be physically active on their own. Program participants were advised to start on an individually suitable exercise level and to focus on the activities that they liked the most.

Participants lived together in groups in small houses with sleeping and cooking facilities, and they cooked their own dinners with provided groceries. Breakfast, lunch, and in-between meals were served in a buffet. The different types of food were marked with labels of energy content. The diet contained 55–60% carbohydrates, 15% protein, and less than 30% fat. At the beginning of the program, each participant learned to calculate their energy intake by counting calories and weighing the food. If necessary, diet plans were adjusted individually according to weight measurements during the program.

The educational program comprised lessons about nutrition, monitoring of food intake and instruction in behavioral techniques from cognitive therapy. The personal development component included a minimum of two individual conversations with one of the psychotherapists, motivational meetings for all participants, and “family meetings” (for all residents in each house) to discuss and resolve problems regarding the living arrangements. In addition, mandatory group therapy classes in personal development were held one hour per week. Classes in personal development were led by a psychotherapist. The goals of this component of the program were to gain insight into personal problems and to consider how these problems may be associated with the obesity. The overall aim of the personal development component was to help the participants live a mentally healthy life as an important part of the process of lifestyle change.

### Participants and Personnel

The participants in this qualitative study also participated in a clinical study investigating the effect of different types of weight-loss interventions, including a stay at the residential weight-loss centre [6]. A total of 30 (21 female and 9 male) Norwegian participants took part and stayed at the centre. These participants were between 22 and 56 years old, their body mass index was between 40 and 63, and the group’s mean body weight was 144 kg. All those who were referred to the program from Norway also participated in a Norwegian effect study [6] in addition to this qualitative study. The inclusion criteria for the effect study were ages between 18 and 60 years old and a BMI over 40 kg/m^2^ or a BMI over 35 kg/m^2^ including comorbidities. There were no additional inclusion criteria for the qualitative study.

The personnel were recruited among the staff at the centre. Since an important aim was to explore the rationale for the choice of program content and structure, key personnel with responsibility for providing the program were recruited to individual interviews.

### Data Collection

For the purpose of achieving immersion in the field of inquiry, the first author (UD) took part in the program over the three week period and observed and interviewed personnel and participants. A data collection period was predefined to two periods totaling to three weeks and was conducted during the 1^st^, 2^nd^, and 14^th^ weeks of the 18-week program. These weeks were chosen to ensure that the initial expectations and experiences (the first two weeks), as well as the experiences from most of the stay (week 14), were captured. During this period, thematic saturation was reached, and no new experiences, discussions, or perceptions emerged in the last interviews or observation.

#### Observation

The first author (UD) participated in all activities as an ordinary participant during the three weeks of data collection, that is, she took part in exercise, classes, and social life. The participants were informed before arrival at the weight-loss camp that the researcher would be present during some of the program, and that this was a part of the larger research project. This period of overt participant observation provided an opportunity to observe real life settings and the interaction between personnel and participants. It also provided insights that were used to inform the development of the question guide that was subsequently used in the focus groups and interviews. The observation also involved informal conversations between the researcher and those personnel and participants who did not later participate in interviews. Field notes were written during observation and at the end of every day. Summaries were developed at several points during the three weeks of data collection. Video and audio recording was not used during observation. The field notes were used to record facts about the program and to write reflections during observations and interviews. Field notes were also used as background for focus group interviews and were later compared with the interview transcripts.

#### Interviews

Those who took part in interviews were asked directly by the first author (UD) and all agreed to participate. A total of 10 Norwegian participants took part in interviews (8 in focus groups and 2 individually). The age and weight range for these 10 persons were the same as for the total sample. Six personnel participated in individual interviews. All interviews were conducted by the first author (UD).

Semi-structured interview guides were employed during focus groups and individual interviews. The guides were used as a memo list to ensure that all the topics were covered in all interviews. The interviewer introduced themes from the interview guide if the participants did not spontaneously talk about them. The participants were also free to talk about issues and topics outside the guide. The main opening question in all interviews was “What are your experiences with taking part in/providing this program?” Additionally, the personnel were asked to describe the content of the program and the rationale for it. Other important topics in interviews with personnel were how personnel attitudes might influence the participants, and how they motivated persons with obesity to change their life-styles.

Each interview lasted from 45 to 90 minutes and was audio taped. All interviews were subsequently transcribed verbatim and de-identified. Redundant words and pauses were removed, and local dialect was changed to written Norwegian.

A strategic sample of eight of the participants, three men and five women, were recruited to focus group interviews. The sampling strategy aimed to achieve some variation in experiences, BMI, age and gender. Focus group participants were interviewed two times, in the 1^st^ and the 14^th^ weeks of the program. The topics discussed in the first interview were immediate perceptions and experiences with the program: overall setting, exercise, living arrangements, and food. The second interviews focused more on the positive and negative experiences that participants reported of the program.

Two female participants were interviewed individually in depth in the first and second weeks of the program. The interviews were conducted to elaborate on specific issues discussed in the focus group interviews. The two participants were selected based on observations of their role in the group of Norwegian participants. During interactions and discussions they had expressed clear views and had provided rich descriptions that offered the potential for meaningful further exploration of key topics in a one-on-one interview. Topics discussed in these interviews included how to make sustainable lifestyle changes, overall approach to weight-loss, and collaboration between the personnel.

Six personnel (2 males and 4 females) took part in individual in-depth interviews. These were considered to be key personnel; the director, the administrative executive, and the leaders of the main areas diet, exercise and personal development. The personnel were selected because they strongly influenced the content and form of the program. Five personnel were interviewed during the first and second weeks. In week 14, three of these were interviewed a second time. In addition, one substitute personnel member was added in week 14 since one of the members had a leave of absence. The second interviews were done to elaborate on the topics discussed in the first interview, and to discuss personnel experiences with the current class of participants.

### Data Analysis

Interview transcripts and fields notes were analysed together. The focus of the analysis was to explore the connections between the personnel rationale for the program and the participant experiences of the program. The analysis was inductive and thematically based. The interview transcripts and field notes were read and the main themes generated. The first and fourth author (UD and AS) read all transcripts and made separate analyses. These analyses were subsequently discussed in a group including the third author (BK), and finally with the second author to get a different point of view and to look for alternative interpretations. It was observed that the personnel articulated several recurrent descriptions or characterisations of people with obesity. These perceptions were described by personnel as forming the basis of the rationale for the program, and so perceptions were employed as a coding structure to categorise and interpret the findings. The validity of the results was cross-checked by re-reading the interviews. To illustrate the main themes the most illustrative and comprehensive quotes were chosen. Quotes were translated from Norwegian to English by the first (UD) and second author (MR) and controlled by the fourth author (AS). Quotes from participants used in the result presentation are identified by interview number and gender. Quotes from personnel are not identified to ensure anonymity.

## Results and Discussion

A total of 10 participants and six personnel participated in interviews. In addition, observation included 30 participants and approximately 10 personnel.

During the analysis of data from interviews and observations, it became clear that there were important differences between how the personnel and participants experienced and perceived the purpose and activities of the weight-loss program. The personnel were very consistent in their perceptions, while the participant experiences and reactions were more diverse. Personnel described very similar rationales for the aims of the program, and for the content and teaching principles used to achieve these aims. Participants had, on the other hand, experiences that ranged from joy and excitement to feeling the program was a struggle and waste of time.

During the analysis, the personnel rationale for the program was categorised into five recurrent themes in their characterization of people with obesity: lack of acknowledgement of resources, lack of structure, a distorted relationship to food, social problems, and lack of personal insight. The program was seen to be intended to change these five characterisations, and the program content and teaching principles were selected deliberately in order to achieve these changes. However, interviews and observation both revealed that participants viewed the program content very differently to the personnel. All participants were very enthusiastic about the exercise program and complied with the strict structures and routines involved in the exercise and diet program. The participants were, however, divided on the usefulness of the parts of the program concerning social and personal development. Division was particularly strong on the usefulness and necessity of the personal development classes.

In the following section, the five characterisations of participants that personnel believed had to be changed in order for participants to achieve and maintain weight-loss, are discussed in more detail. Quotes that describe participant experiences and reactions to the program content are woven into these sections in order to be contrasted with personnel views. Quotes from personnel and participants are used to illustrate and support the findings.

### 1. Lack of Acknowledgement of Resources

The first characterisation ascribed to participants by personnel was the perceived lack of acknowledgement of their own resources in being physically active and sticking to a diet. This was said to be due mainly to low self-efficacy beliefs and low self-confidence. Personnel emphasised the importance of strengthening participants’ self-confidence by ensuring experiences of success in their efforts to exercise and eat properly during the program. Personnel thus highlighted the importance of compulsory and regular exercise and of setting achievable goals.

*The participants shall first and foremost get confirmation that they can do a hundred times more than they believe they can.* […] *It is about transferring the joy of success from one situation to another.* (Personnel).

Participants confirmed this practice by reporting that even small efforts were praised regularly by personnel, and that personnel also frequently encouraged participants individually during group exercise. This was also confirmed during observation. In the interviews, participants confirmed that personnel supported and respected them. This attitude was described as strengthening their motivation, self-confidence, and sense of self-worth.

*That is how they* [the personnel] *build our self-confidence. We are all of a sudden humans again and not just the chubby clown.* (Female, first focus group interview).

When it came to the exercise part of the program participants confirmed personnel claims that success experiences encouraged participants to keep working towards weight-loss. The positive experience of exercise was highlighted both in the observation and interviews from the beginning, and was in fact the most striking finding of the research. The participants were enthusiastic about learning and managing new activities, or activities that they had not engaged in for years or had never tried before. They were also very enthusiastic about improving their physical condition and achieving new goals.

*My first victory* […] *was that I managed to ride a bike for 15 kilometres. I think I walked on air for four days after that experience.* (Female, second focus group interview).

This attitude towards exercise remained stable through the program. In the 14^th^ week of the program, participants unanimously agreed that exercise was fun, increased their energy, and instilled in them a desire to be active in the future.

### 2. Lack of Structure

The second characterisation that personnel attributed to persons with obesity was that they generally lead unstructured lives. Personnel reported a belief that many things in obese persons’ lives were seen to happen by chance, such as when and what to eat, and what activities to engage in.

*Our experience is that many obese people are incredibly unstructured and live extremely unstructured lives. Such as keeping appointments and being stringent and sharp in the things you do.* (Personnel).

According to the personnel, the program schedule had to be very strict. They described that their strictness was important to provide the participants with a daily structure that they could then seek to maintain at home.

*And those who succeed, those we know maintain their weight-loss… they are those who have a structured life afterwards. Those who eat at fixed times and who get out of bed at approximately the same time each day, and those who know what they want with themselves.* (Personnel).

This strict structure was evident across all program activities. All participants had to be present at the morning meetings, group exercises, and at six meals every day (at fixed hours). Other activities, like teaching classes and the weekly weighting, were also scheduled. According to the participants, adhering to the structure was an effort they felt they had to do to comply with the program. Although there were several who compared the program to military training and being back at school with very little freedom, both observation and interviews showed that the participants all agreed that a strict schedule was appropriate to initiate lifestyle change.

*You are supposed to learn some things while you are here. It is like school… subjects are mandatory. And when you know that you have to be ticked off on a list to be registered* [by an employee] *you show up. There’s no alternative. And if it hadn’t been like that the activity would not have been very great. If you are not there at eight o’clock in the morning they call you and ask: “Where are you?”* (Female, Individual interview).

### 3. Distorted Relationship to Food

The third characterisation articulated by personnel proposed that people with obesity have a distorted relationship to food.

*Those with a really high BMI have a behaviour that fully can be compared with an alcoholic or a drug addict. They have no control over food. They always have their form of drug lying around in small storages on secret places so they always know that they are close to the food and they become insecure if it is not in the house.* (Personnel)

According to the personnel, most persons with obesity were addicted to food and would have to live with an eating disorder for the rest of their lives. The program was designed to give the participants the practical experience of eating like a standard weight person and, through that, to change their relationship towards food.

*I tell the participants that they all have a normal weight person inside. They shall hold on to this person and from now on leave the decisions to the standard weight person.* […] *This is not a treatment; it is four months of practice in living as a standard weight person.* (Personnel).

In the beginning of the program, it was observed that participants talked a lot about how to manage the new diet chart, frequency of eating and the amount of food, and the result of their latest weighing. Although there was a consensus among participants about the necessity of managing new eating habits, the experience of enthusiasm and success were less apparent in discussions about diet than about the exercise program. The participants expressed the belief that changing eating habits and adhering to the diet regime was laborious. At the end of the program this was, however, a theme the participants talked less about. When asked, the participants said that following a diet plan had become an automatic behaviour.

*In the beginning I was strictly adhering to the written diet chart, but now I have noticed that I am composing the diet based on my energy target.* (Female, second focus group interview).

### 4. Social Problems

The fourth characterisation of people with obesity highlighted by personnel was a belief that they often have poor social skills. Personnel expressed that persons with obesity often had felt like an outsider and therefore had adopted unfavourable approaches to handle this. Examples of approaches mentioned by the personnel were taking on the comedian role, social isolation, avoiding conflict, and not recognizing or acknowledging their own needs. The housing structure during the program, in which people share apartments and house work and cooking takes place in groups, was deliberately chosen to give the participants practice in socializing with other people.

*Quite a few experience social isolation. So groups are a way to get out of isolation and learn to associate with others.* (Personnel).

The main approach to managing conflict between participants was in line with this focus on social training. Personnel facilitated problem-solving techniques by asking participants what they believed they could do themselves. According to the personnel, this was intended to assist participants with focusing their skills and abilities.

*If two persons share a flat and have different habits… If one of them stays up at night to watch TV and the other person wants to sleep but can’t. Then one is forced to say: “Do you know what, I need to sleep. Could you be so kind as to turn off the TV?”* […] *Then there are two people learning. One is the person who gets the message and the other is the person giving the message. They both learn how it is to enter a conflict.* (Personnel).

The participants had different experiences with the social development component of the program. Some participants described that living closely together was a very positive experience and that they had made friends for life. Others described it as challenging and difficult, and said that they hardly talked with the other participants in their group.

*They have strong emphasis on the mental part, and it is very important… and I feel that I have come a long way* […] *and at the same time… I don’t want to participate so much because… well, there is a lot of gossip here. And if you open up with sensitive stuff you cannot be sure whether it is on every corner afterwards. So I have… I told them* [the personnel] *that this was my decision, and it was fine. This part of the program should have been different. I don’t think all will benefit from it.* (Female, second focus group interview).

### 5. Lack of Personal Insight

The fifth characterisation described by personnel asserted that persons with obesity lack insight into their personal problems. Personnel expressed the belief that lifestyle change require a development in personal insight, and that participants have to reflect on their own behaviour to achieve this. Therefore, the program included classes in personal development. During these group classes, participants were urged to identify underlying factors that may have influenced their obesity problem, such as family relations, working conditions, or economic contexts. The personnel believed such personal development was crucial to enable participants to implement and maintain lifestyle changes.

*It is important that they become aware of what in their life makes a difference in being obese or not. They have some enemies out there and some accomplices* […] *Not only persons… it might also be their work. But to get control over them you first have to be conscious of what they are.* (Personnel).

The participants were clearly divided in their perceptions of the usefulness of the personal development program and views on whether personal development classes should be compulsory. Some strongly disagreed, while others shared personnel views about the importance of personal development. Some participants even asked for more individual sessions with the psychotherapists.

*I think we get tools to gain self-insight and retrieve things we have forgotten… also things that you might not want to touch.* […] *And suddenly* […] *you have changed as a person. And you experience things and perceive things quite differently and you get a different outlook in life.* (Female, second focus group interview).

Other participants saw losing weight, not as a matter of personal development and psychological factors, but rather as a matter of changing bad habits - to eat less and exercise more. These participants were more likely to avoid or refuse to engage in the process of identifying any underlying factors, either by not attending the personal development classes, or by not participating in the discussions during class.

*It is about the personal* [development] *part… I cannot benefit from it. I will never open up in that room and talk among the others. I will not do that.* (Male, second focus group interview).

According to the personnel, those who withdrew from classes or discussions were the persons most in need of attending. Personnel expressed the belief that they were prepared for the type of conflicts that arose, e.g., participants not showing up to classes due to feeling alienated from the content. Personnel explanations of this type of reaction drew on their understandings of some of the potential mechanisms underlying obesity, such as having an eating disorder or a lack of self-insight.

*There are some who do not want to attend our classes. There are some who do anything to avoid them. But they are mandatory. And this is because sometimes you have to hear things more than once before it sticks* […]. *Those who do not bother to attend the lessons are often those who need it the most.* (Personnel).

Personnel perceptions of persons with obesity as persons in need of insight and personal development were noticed by the participants. Some of the participants expressed the view that these generalised characterisations were demeaning, and explained that this could make them feel misunderstood and not treated as individuals.

*…they talk to us like we are all the same.* […] *As if we are flawed.* […] *And as if we all have the same problems.”* (Male, second focus group interview).

### Discussion of Results

According to the participants, the best part of the program was the strict and intensive exercise regime, which was overwhelmingly embraced. Here, the participants and personnel also interacted without any noteworthy conflicts. The participants were very enthusiastic, and the major reason for this was in accordance with the personnel rationale for the program: the experience of success. Experiencing success to strengthen self-confidence and self-efficacy is a well-known approach [26], and such experiences are also recommended to give persons with obesity faith in their own capacity to make healthier choices [27]. Experiencing self-efficacy regarding exercise has also been shown to increase success when trying to lose weight [28]. Experiences of receiving support from personnel were also highlighted by the participants. This is in line with previous findings in which support and encouragement from personnel have been described by persons with obesity as important facilitators for change [29], [30].

The rationale of the program was explained and justified in personnel accounts by articulating a series of claims about the most common characteristics of persons with obesity. The personnel viewed this population as typically resourceful, even if people with obesity didn’t always find this easy to acknowledge themselves, and also lacking in structure, social skills, and personal insight. It was a clear finding that the stereotyping helped the personnel build a joint understanding among themselves regarding both the objectives of and activities in the program. Stereotypes also seemed to help the personnel to remain focused and firm during the program. There are some similarities between the personnel views and the negative attitudes and stigmas that persons with obesity are likely to experience both in society and among health care professionals [31],[32]. Some of the more typical obesity-related stigmas are laziness, non-compliance, and being more overindulgent and less successful than persons without obesity [31], [32]. Persons with obesity have reported experiences of stigmatization on a regular basis, and even more so for those most obese [33]. Inappropriate comments from doctors are in fact among the most common stigmatizing experiences for persons with obesity [34]. Health personnel also attribute obesity to negative characteristics, such as a lack of willpower, sloppiness and laziness [35], lack of discipline, lack of motivation, denial of eating wrongly, and psychological problems [36].

Personnel attitudes observed in the present study might be in conflict with the ideal of building a relationship of trust and acceptance [22]. It would be problematic if the very common negative attitudes towards persons with obesity [33], [34], [37], [38] also exist among the professionals who are supposed to help and support. It is also reasonable to believe that if the personnel perceptions border on stigmatizing, the result for participants will be poor. Obesity stigma has, for instance, been found to negatively influence the motivation to exercise [37] and to increase consumption of calories [34], [39]. Therefore, stigma will most likely not be a facilitator for weight-loss. Personnel in this study are thus likely to walk on a very fine line in perpetuating these ideas for the purpose of supporting participants. One the one hand, it was found that their rationale for the program resembles the same topics as is noticed in the literature on stigma. On the other hand, it was found both in the observation and interviews that the participants for the most part felt that personnel respected them. Therefore, a very clear awareness among personnel also, or especially, at residential weight-loss centres is needed.

According to the personnel in this study, characteristics of persons with obesity were also described as the reasons for being obese, and changing these characteristics was described as a potential way towards a healthier lifestyle. Some studies have shown that persons with obesity agree that determination, commitment, and discipline are necessary to change diet and exercise [29]. To maintain weight-loss, attitudes have been described as important [40], for instance, taking responsibility for one’s life, caring about appearance, self-confidence, and believing that success is possible. Maintaining weight-loss has also been linked to determination, commitment, and patience [41]. Persons with obesity have also confirmed that eating habits can be linked to sadness, lack of motivation, lack of control [29], and negative emotions [42], and obesity has been connected to depression and mood disorders [40]. The mandatory social training in the housing arrangement and the personal development classes in the program were meant to help strengthen participants’ social skills and increase their personal insight. The results showed, however, that some of the participants strongly disagreed with personnel on the usefulness and appropriateness of this approach, especially the personal development classes. Personnel did not see the resistance and objections from some of the participants as an indication that they should rethink and revise the program. Neither did they see the reactions as relating to their own attitudes, but rather as a confirmation that personal development was needed.

Most agree that making changes in exercise and diet is essential to losing weight. The idea that personal development is important to achieve and maintain weight-loss is less well recognised or understood. Claiming that a person with obesity has to change internal factors such as self-knowledge and self-acceptance is also a more sensitive topic than claiming that more exercise and healthy food is necessary to lose weight. Yet personnel argued consistently that personal development was especially important to maintaining lifestyle changes long-term. Studies have also confirmed that knowledge about how to eat healthy is not always sufficient to be able to change eating behaviours long-term [42]. A possible explanation for the participant responses to the different parts of the program could be the length of time between taking part in an activity to experiencing results [43]. While the exercise gave almost immediate results and experiences of success, the area of personal development, with its focus on increasing self-insight to prepare for a lasting lifestyle change, can be seen as more abstract and remote. A focus on the positive outcomes from lifestyle changes are important [44], and the reward for strengthening social skills and gaining personal insight can appear too distant and indistinct.

It could also be important to discuss the criteria of success in weight-loss programs. While successful obesity treatment can be related to absolute weight-loss, another main focus could be to reduce the risk of cardiovascular and metabolic diseases [7]. Although successful long-term weight-loss has been defined as losing 10% of initial weight and maintaining this for one year or more [45], studies have shown that even modest weight-loss can improve psychosocial problems [40]. In addition, moderate changes and the experience of success can motivate persons with obesity to maintain lifestyle changes [46]. This was confirmed by the participants in the present study. Research has, however, also shown that persons with obesity tend to choose immediate rewards over long term benefits [47], and they tend to overestimate the reward from the immediate fulfilment of a need [48]. To wait for long-term reward can be extra challenging, and the connection between the input (personal development classes) and output (weight-loss) would be more challenging, yet imperative, to communicate.

### Strengths and Limitations

The strength of this study is that it is the first to explore and contrast personnel and participant perspectives in a residential weight-loss program that has been reported to be successful [12]. The program has been developed and modified over time according to experiences made in a real-world setting [16]. There are, however, some limitations. The observation took place in 3 out of 18 weeks. A somewhat different picture might have emerged if the whole length of the program had been observed.

The interviews partially revealed situations that were not observed, and in our judgement, we achieved a good overview of the main picture through quite extensive observation and long interviews. However, this study only collected data from a limited group of participants in this program. Although the personnel confirmed that the participants taking part in this study did not differ substantially from other groups, the results could have been different if a different group was studied.

## Conclusion

Personnel characterisation of persons with obesity as a rationale for the design and delivery of a weight-loss program could lead to stigmatizing and discrimination, and thereby less adherence to the program. Participants embraced and adapted to the exercise part of the program. However, the personnel claim that social training and personal development is necessary to lose weight and to maintain weight was not supported by all participants. The social and personal development part of the program is therefore likely to be the most challenging and controversial part of residential weight-loss programs.
